# Assessing the role of non-state actors in health service delivery and health system resilience in Myanmar

**DOI:** 10.1186/s12939-024-02292-3

**Published:** 2024-10-24

**Authors:** K. Than, Maria Paola Bertone, T. La, Sophie Witter

**Affiliations:** 1 Rebuild for Resilience Research Consortium, Edinburgh, UK; 2 Institute for Global Health and Development, Queen Margaret University, Edinburgh, UK

**Keywords:** Non-state actors, Health service delivery, Non-governmental organisations, Ethnic health organisations, Myanmar, Health system resilience

## Abstract

**Background:**

Due to the weaknesses of the public health system and its low reach, especially in border areas, provision of health services by non-state actors (NSAs) has historically played an important role in Myanmar. NSAs include local and international NGOs and civil society organisations (CSOs), but also Ethnic Health Organisations (EHOs) in the border areas, as well as the private (for profit) sector. This study aims to understand the changing role of NSAs in the shifting political environment of Myanmar between 2010 and 2022, and to explore their contribution to health system resilience.

**Methods:**

Our study includes three main components: a documentary review (*n* = 22), key informant interviews (KIIs) at central level (*n* = 14) and two township-level case studies (13 KIIs, 4 FGDs). Mostly qualitative data were collected in 2022 and synthesized, using a health system resilience framework to structure the analysis.

**Results:**

During the transition period (2010–2014) and the new political era (2015–2020), while the country gradually transitioned to a democratic system, the government increasingly recognized NSAs. Initially, engagement with NSAs remained focused on disease-specific activities and government oversight was limited, but later it expanded to health system strengthening, including the start of a “convergence” with ethnic health systems. Progress was relatively slow, but defined by a clear vision and plans. The military coup of February 2021 brought a halt to this progress. Collaboration between government and NSAs was interrupted, and NSAs restored previous practices and parallel systems. Initially, most health service provision stopped, but with time coping strategies emerged, which showed the capacity of NSAs to absorb the shocks (focusing on basic services; using informal communication channels; maintaining buffer stocks of supplies) and adapt (changing modes of delivery and supply chains, and adjusting HRH training).

**Conclusions:**

The study highlights the role of NSAs during crises, and provides insights on how the resilience capacities built over time by NSAs to provide services in adverse circumstances have informed the response to the latest crisis. While strategies of absorption and adaptation are noted in the study, we did not identify any transformation strategy – which might indicate the difficulty of NSAs to introduce radical changes when subjected to multiple shocks and a hostile political environment.

**Supplementary Information:**

The online version contains supplementary material available at 10.1186/s12939-024-02292-3.

## Introduction

As the number of crises and shocks grows globally, and crises become prolonged and shocks multiple, the task of providing adequate healthcare in shock-prone settings is ever more challenging. However, it remains critical to ensure the health of increasingly large and vulnerable communities worldwide. Due to the absence or weakness of the state provision of services in such settings, there often is a proliferation of diverse non-state actors (NSAs), which fill the healthcare arena [1]. The range of NSAs that contribute to healthcare provision varies widely across settings and includes, for example, the private for-profit and non-for-profit sectors, and formal and informal providers (whether qualified or not).

Despite the acknowledgement of their existence, NSAs have been seen as somewhat at the fringes of health systems [1]. Policies and strategies often focus on state-delivered services as governments struggle to find appropriate ways to engage and work with NSAs [2–4]. As such, NSAs and their potential contribution to health system resilience in fragile and shock-prone settings has so far been overlooked. However, in reality the share of service delivery by NSAs (profit and not-for-profit) can be very high in fragile settings [5], and we hypothesise it to be increasing during phases of acute crisis when the state becomes weaker and less capable of effective regulation and oversight. The pluralism of the health sector and the diversity of healthcare providers in crisis-affected contexts can pose challenges, in terms of equity, accessibility, financial protection and quality of the services provided. On the other hand, it has potential advantages and can be seen as a mechanism of resilience, as it can ensure some level of healthcare provision in disrupted times [6].

In order to broaden our understanding of the role of NSAs in fragile settings and reflect on NSAs’ contribution to health system resilience during crisis, this study explores the case of Myanmar, looking at the politically turbulent period between 2010 and 2022.

### Study setting

Since gaining independence from the British in 1948, Myanmar has been challenged by social and political unrest, military dictatorships and civil wars, involving its numerous ethnic groups. After a brief democratic period after independence, the country was under military dictatorship from 1962 to 1988, and again from 1988 to 2010, during which the country was mostly closed to international relations [7] and the central government was in conflict with autonomous border areas. In the wake of cyclone Nargis, which hit Myanmar in 2008 causing over 130,000 deaths and leaving millions of people homeless, international assistance arrived in Myanmar and this external support and the gradual engagement of the international community in Myanmar played a role in the establishment of the quasi-civilian government in 2010 [8]. The new government opened the country internationally and started negotiating ceasefires with Ethnic Armed Organisations (EAOs) active in the border areas, with the aim of improving the socio-economic status of the population by strengthening service provision, including in border areas [9]. However, the country was struck again by multiple crises in 2020–2021 when the COVID-19 pandemic hit the country, and a coup on 2 February 2021 started another period of military dictatorship.

As a consequence of this history, before the democratic transition of 2010, the public health system in Myanmar was weak and had low reach, especially in hard-to-reach, border and marginalised areas which have been long under the control of EAOs. The public healthcare infrastructure had remained patchy and the public health workforce limited in numbers and unevenly distributed [10, 11]. The health system was underfunded by the government, with public health expenditure at below 1% of the GDP until 2010 [12], as well as by external actors due to the sanctions against the Myanmar government. In contrast, provision of health services by non-state actors (NSAs) has always played a significant role in Myanmar. Indeed, most of the external funding has been historically channelled to non-state health organisations, with a particular focus on the conflict-affected areas around the South-East border [13].

NSAs in Myanmar include a diverse range of actors, which has evolved over time due to the weakness of the state and support of external actors, and to respond to the needs of the communities they served. Civil society organisations (CSOs) and local non-governmental organisations (NGOs) have their origins in village-level religious organisations that historically gathered people for social or religious activities [14]. CSOs became more formally established in the early 1900s, and by the end of the 20th century a clearer political and/or ethnic dimension in terms of their affiliation emerged. In the 2000s, CSOs and local NGOs flourished in both central Myanmar and ethnic-controlled areas, due to worsening socio-economic conditions and the lack of basic services provision by the government, and began directly providing provide health and other services to meet community needs [15]. In parallel, the presence of health-service providing international NGOs increased in particular in response to Cyclone Nargis (2008) and later with the democratic transition (2010) [15]. The role of INGOs has been of directly providing health services, but most often of channelling funds between donors and local NGOs or service providers [16]. In 2021, there were 63 NGOs and INGOs working in the health sector (i.e., 40% of the total NGOs in the country) [17].

In addition, Ethnic Health Organizations (EHOs) provided health services in Myanmar, with a focus on the border areas (southeast Myanmar, Kachin and Rakhine) which had historically remained outside the government’s full control. Many of the EHOs were established by Ethnic Armed Organizations (EAOs) to address gaps in health service provision and fulfil the health needs of populations in the border areas. Overtime, they developed their own health systems [18]. With financial and technical assistance from international aid through INGOs and expatriates or diaspora (often with funding flowing through the Thai side of the border rather than through Myanmar via Yangon), EHOs developed standard clinical protocols and training curricula for health workers to build capacity of their own human resources, created an infrastructure network as well as established a supply chain to purchase and distribute medicines and commodities through various channels. Usually, services provided by EHOs covered primary health care in clinics and through mobile clinics or teams, often making use of community health workers or village staff for outreach. Services remained free of charge. In 2017, there were more than 30 EHOs in Myanmar’s border areas [18].

Finally, the private sector played an important role in health care delivery in Myanmar. The economic liberalisation reforms of 1988 resulted in the growth of the private health sector [19], which started with small private clinics and grew to include specialist clinics and hospitals in urban areas. However, individual private general clinics run by medical doctors, known as General Practitioners (GPs), have remained popular over time. They are often the first point of care for the general population in the communities, and can be found in both urban and rural settings [20]. The private-for-profit sector also includes drug importers, pharmacists, and a large and growing number of medical practitioners working exclusively or primarily in private practice, as dual practice is common [21]. In the late 90s, social franchising programmes with partnership between non-profit for-profit clinics also started developing, in particular focusing on services such as family planning [22]. According to the Private Health Statistics 2015 by the Department of Medical Services, in Myanmar there were 193 private hospitals, 201 private specialist clinics, 3,911 private general clinics, and 776 private dental clinics [23].

### Study aims

This study aims to explore the role of NSAs in Myanmar and how it has evolved and changed over time as the country navigated different shocks and crises. We focus on the period starting in 2010, which provides background for a more granular understanding of the current situation and how the role and features of NSAs, developed in past decades, have shaped their current role (after the 2021 military coup). The historical analysis highlights the resilience capacities built by NSAs as they navigated previous crises and how these were used to support coping strategies post-2021. Such analysis allows for a broader reflection of the contribution of NSAs to the resilience of the health system in Myanmar.

## Methods

### Study design

The study adopted a descriptive multi-level design, as it included data collected at central level and also two township-level case studies. The study had both retrospective (2015–2020) and prospective (2021–2022) elements, and drew mostly on qualitative data. Primary data were collected between December 2022 and March 2023.

### Sites for case studies

The two case study sites were initially meant to represent areas that had different histories in terms of previous shocks and crises, and the role of NSAs, including one central and one ethnic/border area. However, due to security concerns, the choice was eventually focused on the central site and another in the Southern part of Myanmar. For security reasons, we refer to them as township 1 and 2.

Township 1 is a peri-urban township in Yangon, with a dozen government health facilities including hospitals and health centres, and numerous private clinics, NGOs and CBOs [24]. The township has a large migrant population working in factories, and was one of the township with high COVID-19 cases [25]. Following the 2021 coup, many individuals and communities in the township have been involved with the Civil Disobedience Movement (CDM) and the political uprising. This has led to high levels of political and social instability with numerous detentions, which has been one of the areas where marshal law has been imposed.

Township 2 is in the centre of the country. It has large population, including both urban settings and rural villages. In terms of health networks, there are numerous government facilities and some private clinics, CSOs and NGOs [24]. Many in the township participated in the CDM and as a consequence the area is strictly controlled by the government, with tight rules restricting movements (for example, two males are not allowed to ride on a motorbike, the most common transportation mode).

### Data sources and data collection

There were three main data sources for this study: (i) document review, (ii) key informant interviews at central and township levels, and (iii) focus group discussions (FGDs) at township level.

A review of the published literature and grey documentation was carried out, including academic papers and documents such as reports, government and district level policies, international partner reports, plans, guidelines, and programmes. Particular attention was given to capture the historical aspects and changes over time in the role of non-state providers, as well as their relations to state and international actors. For the review of published academic material, a search was conducted through the online databases PubMed and Google Scholar. Key search terms were utilised to return relevant documents in terms of geography and sectoral focus e.g. “Myanmar” OR “Burma”, “health” OR “health service provision”, “Non State Actors” OR “Non-Government Organizations” OR “Community Based Organization” OR “Ethnic Health Organizations”. For grey literature, purposive screening, identification and listing of sources was conducted before a search was conducted for relevant documents and terms. The document collection process was iterative and only relevant documents were included for data extraction and analysis, after screening. A total of 22 documents was included.

A series of key informant interviews (KIIs) and focus group discussions (FGDs) were carried out at central and township levels. Participants were chosen to represent different stakeholders, including different type of NSAs, and because of their knowledge or experience of the health system at national or township level as implementers, providers or service users. Due to the funder’s no-engagement policy, no government representative was included in data collection. Sampling of KIs was purposeful, with the aim of being representative of actors involved in non-state provision of health services in Myanmar. Participants were contacted via email, telephone or in person to explain the purpose of the study and propose the interview, and those who agreed to participate were asked to read and sign an information sheet and consent form (consent was taken verbally in the case of remote interviews). Interviews were carried out in English and Burmese depending on the preference of the interviewee. Interview lasted around one hour and were recorded. Recordings were then transcribed and translated into English where necessary. Topic guides were used during KIIs and FGDs which were adapted to the specific level of the informants (Annex 1).

At central level, topic guides focused on understanding the role of the NSAs in the health sector in the period between 2018 and 2022, enablers and barriers in carrying out health service delivery by non-state actors, and how this contributed to the overall resilience of the health system (e.g., through which pathways and by developing or not which resilience capacities). At township level, KIIs targeted health providers in the NSAs and focused on the post-2021 period, with questions on how provision of health services had been adapted in the politically unstable context and how challenges in different aspect of the health system were addressed by providers. Finally, FGDs adopted a participatory approach and consisted of two sessions. First, participants were asked to list all providers available in their area. Providers were subsequently categorised by type (public/governmental, NGOs, CSOs, etc.) and participants ranked them according to usage (most to least commonly used) during different key periods in relation to the COVID-19 and the insecurity phases faced by the country since 2021. Secondly, questions were asked for each category of provider in terms of quality, accessibility and satisfaction of service users and questions to explore healthcare delivery and health seeking behaviour were asked by the researchers.

Fourteen KIIs were carried out with actors at central level. Table 1 below provides an overview of the categories of KIs that were included.

**Table 1:** Summary of KIIs at central level

Category	Sample
International donors and funders	6
International NGOs	3
Local NGOs	3
EHOs	1
Private sector	1
**Total**	**14**

At township level, a total of 12 KIIs and 4 Focus Group Discussions (FGDs) were carried out with a range of stakeholders including NSA providers and service users, as outlined in Table 2 below.

**Table 2:** Summary of KIIs and FGDs at township level

Site	Category	Data collection approach	Sample
Township 1	NGOs	KII	2
	CBOs	KII	1
	Community-based service providers	FGD	1 FGD with 10 participants
	Service users	FGD	1 FGD with 10 participants
Township 2	CBOs	KII	2
	Private sector	KII	2
	International NGO	KII	3
	Local NGO	KII	2
	Community-based service providers	FGD	1 FGD with 8 participants
	Service users	FGD	1 FGD with 11 participants
**Total**			**12 KIIs** **4 FGDs (with 39 participants)**

### Data analysis and synthesis

Data analysis was conducted separately for each data source. Documents were analysed based on content analysis, working deductively and inductively from the research questions. Data extraction was carried out manually, comprising preliminary identified themes and codes, for example type of organisation or actor, type of service provided, funding source, or modes of delivery. Interviews and FGDs were analysed using a thematic framework approach which facilitates rigorous and transparent analysis and uses both deductive and inductive approaches [26], working from the initial structure of the resilience framework [27], but adding codes as needed.

At a second stage, findings from different data sources were brought together, triangulating, comparing and contrasting the emerging results and describing the overall findings of the study. The findings are organised mainly in chronological order to show the importance of the role, engagement and learnings of NSAs in previous phases to inform the strategies adopted in response to the current crises. To discuss and reflect on the findings, we adopt a framework focused around the concept of health system resilience. By health system resilience, in line with recent literature and conceptualisation [28], we refer to the health system’s ability to maintain core functions and minimise the negative consequences of sudden shocks or stressors, by adopting coping (or resilience) strategies in order to absorb, adapt and/or transform in face of such disruptions. Drawing on recent work [29, 30], we define the three main categories of resilience strategies as:

- Absorption: system’s ability to respond to population needs using available resources and organizational processes.
- Adaptation: system’s ability to adjust how its resources operate without changing system structures.
- Transformation: when needed, the system is able to change its structure, organisational processes and the way it uses resources to address population needs, both pre-existing and new.

**Fig. 1:**
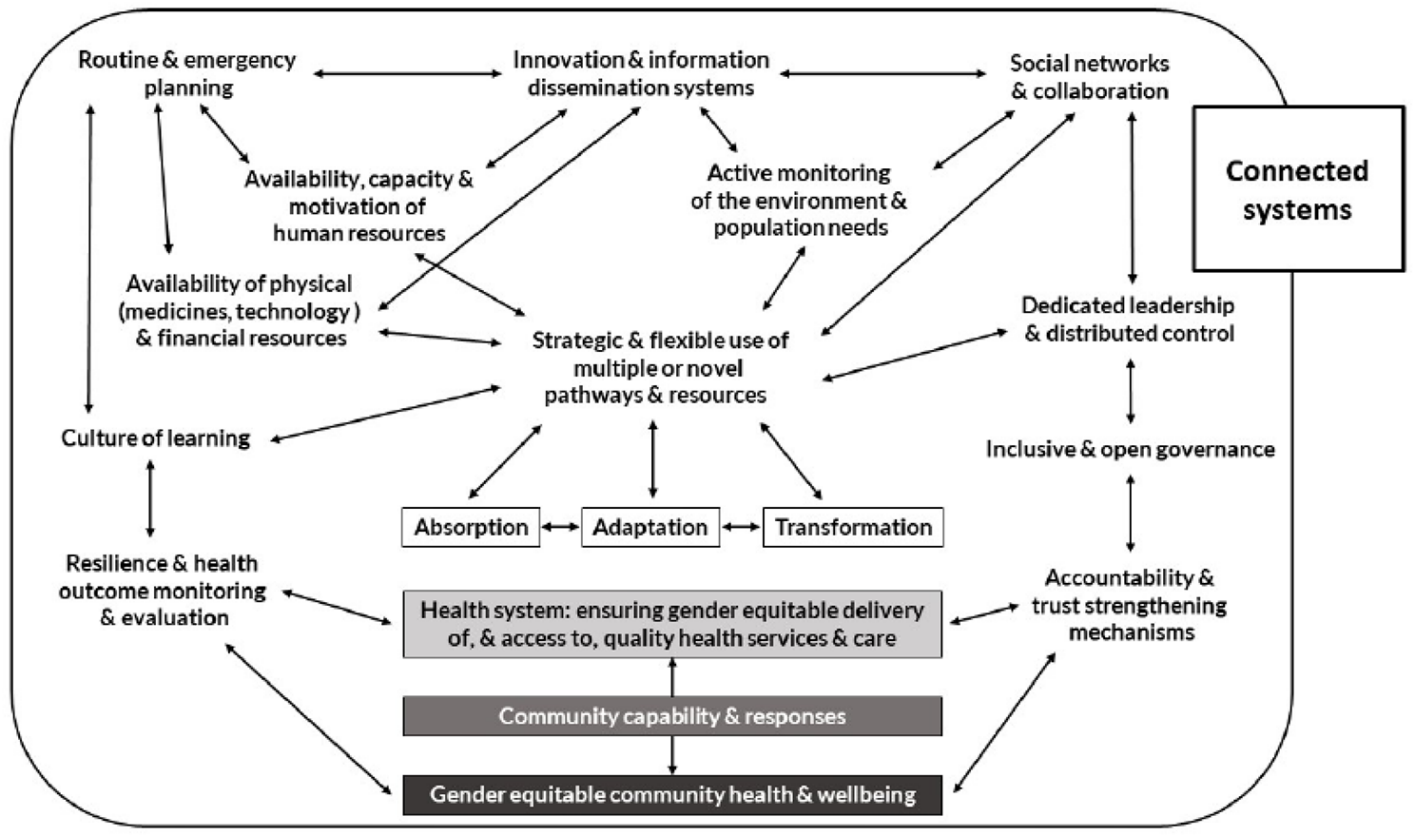
R4R Resilience framework. Source: [27]

Specifically, we adopt the ReBUILD for Resilience (R4R) Resilience Framework (Fig. 1) which conceptualises absorption, adaptation and transformation as dynamic processes or strategies. Importantly, the framework discusses absorptive, adaptive and transformative (resilience) capacities. These are the underlying broader capacities that the health system must have in place in order to deploy any of the resilience strategies. Resilience capacities refer to both specific elements (e.g., the presence of a culture of learning within the health system) as well as the interlinkages between elements [27].

### Ethical considerations

for the study were obtained from the Liverpool School of Tropical Medicine (LSTM) in the UK on 17 February 2022 (research protocol number: 21–082).

Care was taken to ensure the safety of all participants as well as researchers despite the challenging security context. A flexible approach was taken to data collection, allowing for remote interviews were needed and changes in timing and location to township-level work. Ensuring anonymity of participants and confidentiality of the information they shared was an essential consideration throughout the study. Transcripts were anonymised as soon as possible after transcription and only anonymised data was used for the analysis and shared between teams. In the write up, we assign quotes to participants using their category in a way that ensures the preservation of anonymity. Additionally, we have opted for not disclosing the location or study site from which some of our quotes or findings emerge.

### Findings

**Fig. 2:**
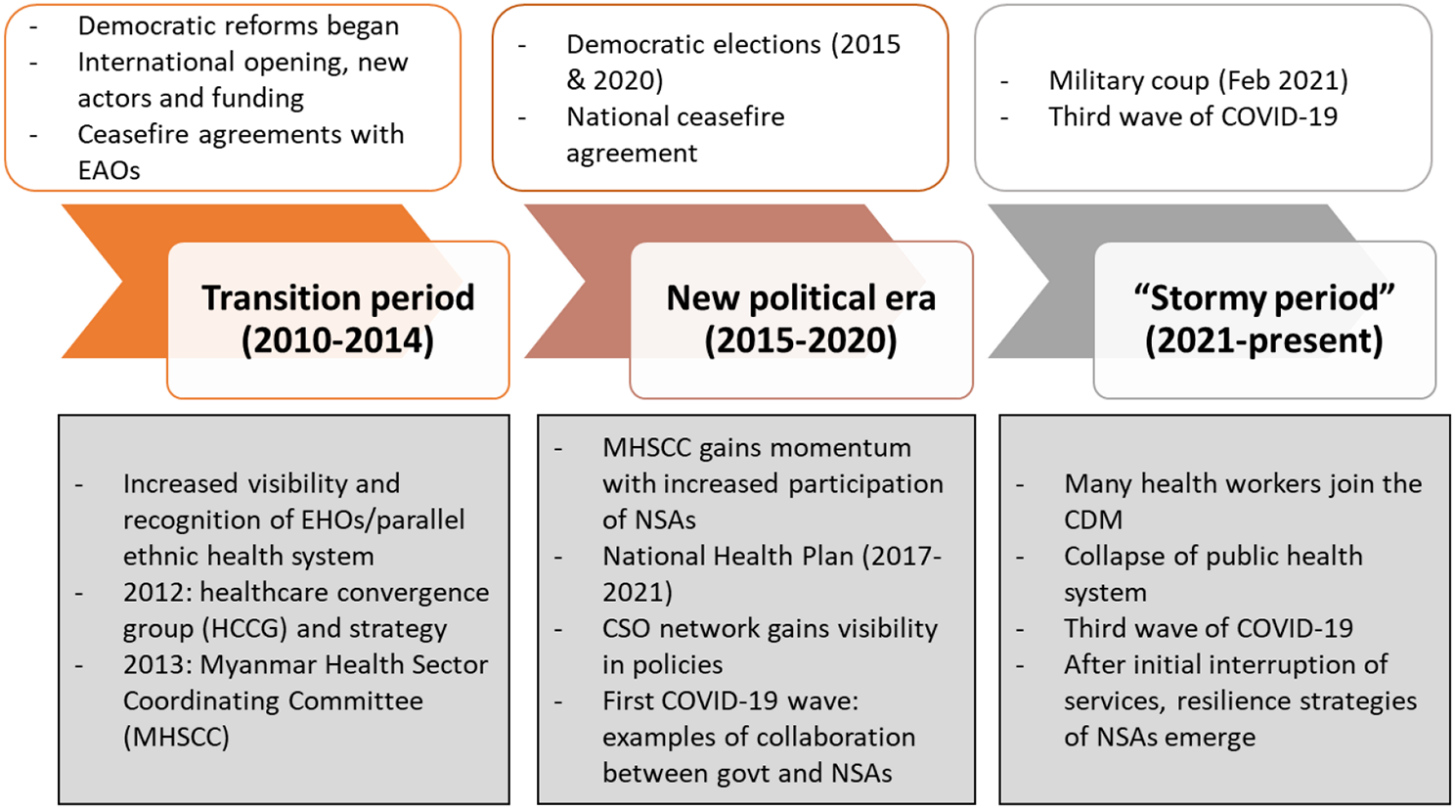
Timeline of key periods for the health system in Myanmar. Source: authors

Findings in this section are presented along a timeline (Fig. 2), which identifies the key periods, starting in 2010, in relation to the political environment and the health system. For each, we describe the main features of NSA engagement and approaches. For the most recent period (after February 2021), we also reflect on the coping mechanisms and resilience strategies, both as recounted as central level and in the case study settings. While information for past periods is mostly based on the documentary review, the findings on the current phase (2021 up to now) and the case studies are based on our primary data collection.

### NSAs during the transition period (2010–2014)

From 2010, the opening of the government to democratic competition set the stage for a transition period, which established the foundations of the Myanmar health system over the following 10 years [31]. New external actors started operating and new funding flowed to Myanmar for the provision of health services, including in the border areas and hard-to-reach areas, which were previously almost completely excluded from public health provision [32]. In 2013, the Myanmar Health Sector Coordination Committee (MHSCC) was formed, chaired by the Ministry of Health and Sports (MoHS) and with the Global Fund (which had restarted operations in 2011, after interrupting them in 2005 and was Myanmar largest donor) as the secretariat. Importantly, other members included NSAs, such as NGOs and Community and Faith-Based Organizations [33]. This more formalised role for NSAs was in line with the political environment, which since the 2010 general elections had allowed more political space, prominence and rights to CSOs, including in the negotiation of the ceasefire with EAOs. From the health sector perspective, the ceasefire negotiations brought visibility to the parallel ethnic health systems, especially in the Karen states, which had been receiving direct funding from international donors for decades [34]. While “convergence” between EHOs and public health system started being discussed in 2012 also as a tool for state legitimisation in the border areas [13], in practice EHOs continued to operate under the management of EAOs, and therefore beyond the control and the guidelines and standards of the central government (for example, in terms of staff training), continuing to rely on donor support for their funding [35].

In general, despite the opening in the political environment, in the 2010–2015 period the engagement of government with NSAs remained relatively minimal and limited to disease-focused activities (rather than broader health system strengthening). Regulatory frameworks and government oversight were also weak and limited so that the quality of care across NSA providers varied significantly [36]. NSAs continued to play an important role in relation to service provision for underserved population, filling the gap as public services were still lacking in many areas.

### NSAs in the new political era (2015–2020)

The 2015 electoral victory for the National League for Democracy (NLD) and again the democratic outcome of the 2020 elections meant further progress for the Myanmar health system. The MHSCC emerged as more effective and active and oversaw an improvement in coordination in the health sector and better inclusion and dialogue with civil society organisations (CSOs), international and local NGOs, as well as EHOs [33]. Reflecting on the progress made on health indicators across Myanmar during the period (and despite the challenges of the first waves of the COVID epidemic towards the end), the strong commitment and collaboration among the implementing partners of NGOs, INGOs and UN agencies emerged as one of key factors for these achievements. One of our interviewees called this period the “golden years” and said:

*“*I would call them really the golden years for the Myanmar health system. That period of course was the NLD time when health and social services were prioritized. So let me paraphrase it. It was the fairest democratic government that built into the previous transition*”* (KII-004; international donor; central level).

We describe here some of the noteworthy changes and shifts in strategies, approaches and regulation which affected how NSAs operated within the health system of Myanmar.

Starting in 2015, it became legal for INGOs to work with EHOs while being based in Myanmar (before support was provided from Thailand or elsewhere). However, the “convergence” between health systems set up by the EHOs and the governmental health system remained politically sensitive. In 2014, the newly created Health Convergence Core Group (HCCG) which assembled most EHOs, had developed a convergence model which called for EHOs to remain in place and argued for support to a federal decentralized health system, rather than simply strengthening the central government system [37]. While the government recognised the need and benefits of collaboration and (broadly) the principles of convergence, the vision on the process was different on the MoHS side, which envisaged a more top-down integration rather than the power-sharing model proposed by the HSSG [35]. Despite divergence of principles, when the Nationwide Ceasefire Agreement (NCA) was signed in 2015, progressive collaboration occurred between the two health systems. Examples of successful convergence activities included information sharing and trust-building seminars, joint trainings, joint immunization programmes in Kayin and Kayah, and joint response to a cholera outbreak in 2015 [13].

On the other hand, at this time, government oversight and regulation of NSAs increased, reducing the levels of flexibility and independence that they had before. For example, INGOs and local NGOs started being required to sign a Memorandum of Understanding (MOU) or Letter of Agreement (LOA) with the MoHS to implement approved health services in clearly defined geographical areas. The MoHS also monitored, supervised and provided technical and clinical guidelines for INGOs and local NGOs, which were required to report to the MoHS. Likewise, private clinics and hospitals required a licence from the MoHS to legally provide clinical services.

*“*During this period, the MoHS was leading most of the TWGs [Technical Working Groups] and discussions were more open. We were able to share where we work and how we work. Standardizations of clinical guidelines happened more quickly and efficiently*”* (KII-003, International NGO, Central level).

While the new regulations allowed for increased standardisation of health service provision, avoidance of duplication and generally better governance and stewardship of the health sector by the MoHS, it also limited the adaptability of NSAs in relation to service provision, for example with reference to modes of delivery and access to some of the areas in the country, as well as ways of responding to localised challenges.

By the end of 2016, the comprehensive National Health Plan (NHP 2017–2021) [38] was launched, which acknowledged the role of NSAs, not only in the strategy-drafting process but also as essential to deliver PHC in the country in order to meet the minimum standards in delivery of health services to the people in hard-to-reach areas [38]. Specifically, the NHP’s vision was that of developing the MoHS as the purchaser of services via the establishment of a semi-autonomous purchasing body, to contract with and purchase services included in the Essential Package of Health Services (EPHS) from a range of providers, including NSA providers such as EHOs, CSOs, NGOs and private providers [35, 38]. Funding to purchase health providers outside of the MoHS was planned to be initially provided by donors and development partners, and only later transferred back to the MoHS [35, 39].

The international community continued to explicitly acknowledge the importance of both EHOs and NGOs/CSOs for health service provision in the changing environment of Myanmar, and made commitments to providing technical and funding support to the health system, channelled mostly through NGOs and INGOs [40]. Donors recognised the specific needs of the context, including (as detailed in the key operational principles of the multi-donor Access to Health Fund) that of conflict-sensitivity and social cohesion, as well as flexibility to best deliver on the mission [41].

Under the NHP, the government was also committed to strengthening private sector engagement through the recently established Private Sector (PS) Unit in the MoHS. Supportive policies were in place, including Private Sector and Public-private partnership (PPP) laws, however there were no clear strategies on how to engage the private sector. As data on private healthcare sector in Myanmar was scarce, incomplete and fragmented, the MoHS was unable to fully comprehend its contribution to health service delivery. Government investment in private sector engagement remained limited and mainly focussed on regulation, which was perceived as punitive by private sector actors, while government actors sometimes considered the private sector to be corrupt and unethical. As a key informant from the private sector stated:

*“*It’s really sad we are still struggling with regulations till now and there is a big conceptual gap between inside and outside. We contributed a lot for the people when crisis like COVID happened but they only see us and keep on criticising up as benefiters*”* (KII-014, Private sector, Central level).

In 2020, as the COVID-19 pandemic hit the country, the progress that had been made in Myanmar in terms of strengthening the health system with the contribution of all actors, including NSAs, contributed to the effective response during the first and second waves of COVID-19 (March-June, and August-November 2020). During this period, efforts to contain the virus were achieved in collaboration with NSAs and COVID-19 offered a further test for the cooperation and convergence between EHOs and government health services, with positive results [34, 42].

*“*During this period, all sectors of the health [system] were starting to improve, many of the donors like the World Bank, ADP, Access to Health Fund and Global Fund were more focused towards overall system strengthening. […] Fund flows came in [the country] from many places and the health sector was growing with a momentum*”* (KII-002, International NGO, Central level).

Despite the vision and plans and although there was clear progress in collaboration between state, non-state and external actors, as well as the improvements in standardization and regulation, progress was generally slow. For example, the convergence process remained largely unfinished and disputed. The main challenge identified in a 2020 study was that convergence was inextricably linked with the political and peace process, which never reached a full peace accord, along with other challenges such as lack of trust between key stakeholders, the centralised nature of the MoHS, the lack of accreditation for EHO’s health workers, and the weak implementation of the NHP in some ethnic areas [35]. In practice, most NSAs continued to provide health services with the approaches (including delivery modes, standards, guidelines and regulations, supply chains and funding flows) that they had developed over time to suit their context and needs, and many challenges persisted.

### NSAs during the “stormy period” (2021-present)

The military coup of February 2021 brought a sudden halt to the progress made in the previous 10 years in terms of health system strengthening, integration of NSAs and dialogue between NSAs and government. As described by the New York Times [43], the “Myanmar’s health system [was] in collapse, obliterated by the regime”, while having to deal with the third wave of the COVID-19 pandemic [25]. The combination of the military rule and the COVID restrictions meant challenges such as travel restrictions, lockdowns, road blockages, limitations on mass gatherings, supply chain blockages, suspended activities, training and events, and the re-prioritization of health staff away from essential routine activities towards COVID-19 response.

### Immediate aftermath of the coup

Immediately after the coup, NSAs’ health service provision came to a pause, with few exceptions focusing on humanitarian response. NSAs faced multiple challenges, including having to navigate the funding landscape which changed overnight as donors withdrew funding from the country to avoid subsidizing the military government. Due to the interruption of any collaboration between NSAs and government, NSAs had to maintain a low profile. In addition, many NSAs opted for a no engagement policy with the military government to avoid social punishment as most communities were against the rulers, and also to limit security risks.

*“*Organizations were caught between non-engagement on one hand and social punish on the other. Working for the people and to reach the people was our utmost importance and we continued activities, maintaining a low profile” (KII-002, International NGO, Central level).

In the tense political environment, communications and coordination channels collapsed, and there was minimal information sharing as NSAs found it difficult to know who was reliable and re-establish trust relations.

At the same time, the public health system had de facto collapsed due to the crisis, compounded by many health workers joining the civil disobedience movement (CDM) and leaving their position in public facilities. As estimated by participants in the study, only about one third of the health workers in government sector were left in the system and many of the hospitals were closed due to shortages of human resources for health.

*“*Most of the government facilities are closed especially in the rural areas as there is no one left. Even in the large cities, hospitals were occupied by the military with the reason identified as security. It is really hard for the people in the community, especially with no money, they have nowhere to go in case of emergency*”* (FGD Township 2).

Essential services, which were primarily run by the government, such as immunization campaigns, stopped. Against the backdrop of the collapsed public health system, the contribution of NSAs became even more essential as they slowly restarted to provide basic health services. As described in the following sections, after the initial shock, health-service providing NSAs had to quickly revert to previous practices and parallel systems. The experience in dealing with a complex environment in past periods and the strong linkages with the communities they served laid the foundation for the coping strategies implemented by NSAs in this most recent crisis. As one of the key informants stated,

*“*There was no easy way working during this period, we had to struggle so much but, I really find the collective resilience which helped us reach the affected and real need communities through trust and local commitment, taking high risk of personal security*”* (KII-007, Local NGO, Central level).

### Absorbing the shock

The strategies implemented initially by NSAs focused on absorbing the shock, whilst maintaining minimal service delivery and safety for their staff. NSAs continued service delivery although with a reduced focus, targeting TB, HIV and MCH services, which had historically represented the core of their engagement. In addition, the modes of delivery were adapted to the context and to the health seeking pathways of the patients (which were also constrained by the new context). For example, private GPs became the first line of contact for emergency care for the community, due to the closure of hospitals and in some cases due to the risk of accessing government-affiliated public hospitals.

Supply chains were interrupted for some time, but many NSA providers had maintained buffer stocks – a practice that had remained from previous crises. While communication and coordination had stopped in the immediate aftermath of the coup, informal coordination mechanisms between NSAs started again gradually and with a low profile. However, trust remained a key issue initially, especially for health staff who was being targeted by the military. As one interviewee said:

*“*Informal networks of doctors and nurses are still working, donations come in very secretively with no identification of donors and we work only with a close knit of people who can be trusted and know each other, we give service not only in the cities but reaching as far as we could*”* (KII-007, Local NGO, Central level).

In this context, ensuring the safety of the staff was of paramount importance for NSAs and provision of health services had to be adapted to ensure safety. The use of community-based, mobile volunteers which had been in place during previous crises and could now have a renewed role was one of the immediate coping mechanisms adopted to maintain service delivery:

*“*As the conditions get worse, we had to start protecting our own staff for COVID as well as not to get caught: safety was first-priority. We just told them to look at the ground situation, if need to close, we close and we give drugs through local volunteers’ backdoor*”* (KII-003, International NGO, Central level).

### Adapting to the new context

With time, NSAs’ coping strategies focused on adapting their approaches in a more systematic way. Adaptations were introduced in **service delivery arrangements**, in order to reach the most in need and adapt to the local situation and the ever-changing context. This required the strong grounding in the local communities which NSAs had built during their long engagement. To reduce risks, tasks were shifted as much as possible to volunteers or members of the patients’ families, and health service provision was coupled with supply of other humanitarian goods, such as food and clothing so to make it less prominent as health activities were often targeted by the military. Clinical practice such as length of prescriptions changed, becoming monthly rather than weekly to avoid multiple visits by patients. In some cases, opening hours were modified or even physical offices were closed and phone consultations introduced to reduce the need of movement and any potential targeting of staff, patients and facilities by the military. In other instances, evidence from the case studies showed that NSA facilities expanded the opening hours and services in order to cope with the increase in consultations due to the failures of the public health system. Patient referral between facilities became difficult because of the breakdown in communication and collaboration between public and non-public sectors. However, participants mentioned that, despite the difficult environment, they were able to connect with and ask for assistance to other providers (including public), by doing so informally, building on their previous relationships.

With the international **supply chains** not available any more, maintaining availability of essential drugs and supplies was a major challenge for many NSAs. NSAs reverted to the local purchasing of required essential commodities. NSAs also collaborated through informal pathways to ensure supplies in the areas where they were needed most. These strategies were grounded in the capacity built in previous periods, also by the support of donors in terms of strengthening of the private sector contribution to supply chains.

*“*In the early phase we used buffer stocks for medicines, but we also had good coordination between one another. Since the supplies were hard to obtain, we used a local purchasing mechanism. However, as you know the restrictions are much tighter now and I do worry about essential medicines and supplies*”* (KII-003, International NGO, Central level).

In terms of **health workforce**, there was a shift in their distribution due to the adherence of many health staff to the CDM. CDM staff who were working for public hospitals and often also having a second practice in the private sector stopped working in their public employment. This resulted in shortages of staff in some areas and for some providers. To cope with this, various strategies were implemented, including task shifting and involving family members to support healthcare provision. Additionally, staff seconded from NGOs became essential for the provision of certain services such as national disease programmes in the absence of sufficient government staff. One responded in Township 2 mentioned,

*“*Later [after February 2021], most team leaders from MOH were not there because they engaged in CDM, so NGO staff, the seconded staff, had to run the services*”* (KII-021, Local NGO, Township 2).

On the other hand, CMD workers who moved to border areas were able to support health service delivery there. Skills-building initiatives were introduced in the border areas where skilled CDM workers had been displaced and could support capacity-building efforts, which were instrumental to the establishment of new secondary care facilities in those areas.

**Funding** for healthcare was a main challenge especially as existing funds started to dwindle. For safety reasons, sources of funding were kept as secret as possible, and reporting started operating in an oral way, based on trust rather than on written documentation that bore high risks. The diaspora played an important role in supporting funding flows and, coupled with the expertise of CDM specialist doctors in border areas that helped to build local health systems for emergency care services for the most vulnerable population.

*“*It’s so hard to send medicines and equipment to the conflict effected areas, we used different mechanisms to reach to those areas, taking risk of human lives and CDM doctors were our visionaries in helping the worst emergency situations*”* (KII-003, International NGO, Central level).

However, banking was a critical bottleneck as it allowed government scrutiny of finances and revealed activities and staff. As a consequence, NSAs found alternative ways to ensure cash flow for their organisations.

To address the funding challenges, donors’ flexibility was an element that was recognised to have supported NSAs and contributed to the continuation of their operations. All donors working in Myanmar had learned over time to navigate the political difficulties, and even when supporting health system strengthening through government/public structures and bodies, they had in parallel continued to reinforce community health systems and kept their funding somewhat parallel through multi-donor funding. This approach proved effective when funding to government was stopped and focus returned to supporting NSAs. Flexibility in approaches had always been a core principle, as highlighted above and also in the empirical literature [34], and this allowed donors to understand and adapt faster, supporting the NSAs’ coping strategies.

*“*Donors tried to understand the ground situation with open ears and were flexible in funding approaches, it was helpful in getting to the hard-to-reach conflict affected areas with local people and local means in a low profile*”* (KII-007, Local NGO, Central level)

Another challenge was to navigate the **engagement with government**. Despite the desire of many NSAs to remain independent of the government and limit interaction, the government through the Ministry of Health attempted to maintain strict control over their activities. This included strengthening the registration and licensing rules that had been introduced in 2014. The aim now was less that of standardisation and coordination between providers, and rather had a focus on tightening control. One respondent said,

*“*They (MOH) are strict on us, and we have to inform them whenever there is anything or any activity. We can go to the field for project activities only if one of them (someone assigned from MOH) with us. So, it is difficult for us. We can’t cut off the communication completely, so we have the least engagement with them but not obviously. If we do, there is also social punishment (from the community/citizens)*”* (KII-021, Local NGO, Township level).

The constraints in terms of funding, security concerns and the additional government restrictions meant that some NSAs and in particular smaller CSOs had to stop providing health services. In one township, for example, participants reported:

“During the third wave of COVID [July – December 2021], CBOs had to disappear because some did not have funds, they needed to ask permission from them (government) to do activities. They can’t do activities openly, many felt depressed, so did not have strength to run (the organizations). Most of the people who are working charity-based activities are not rich, they need to earn daily to feed their family, so the numbers of people who working charity-based activities are reduced” (FGD-04, Township 2).

On the other hand, other type of NSAs were able to continue working. This includes NGOs which were found to be particularly active in the provision of healthcare services in Township 1, despite the challenges faced due to the political context. With people not wanting to go to public hospitals, it was noted that private hospitals increased their operations from 2022 onwards, to address the gap in service provision. However, private services remained costly and affordable only for few patients.

Our findings show that patients also adapted their **health seeking behaviour**. For example, in some border areas they sought (emergency) healthcare across the border, which had been a strategy also implemented in previous times of insecurity and to which communities could revert.

*“*The regions [Karen and Kachin] have been in conflict for centuries and know how to navigate and get emergency care, but for inland closed regions like Saggaing, I think it is more difficult to reach emergency health care services*”* (KII-004, International donor, Central level).

In other areas, patients changed their preferences in terms of providers, switching to what was available (which had changed, especially due to closure of public facilities) and affordable, with less attention to quality and more to security – for example, by limiting distance and movement and avoiding facilities close to military installations or closely controlled by the military.

*“*I just go to the nearby health worker in my village, I dare not go to hospital where it’s too close to the military base*”* (FGD-03, Township 2).

## Discussion

In this section, we provide an overview of the key findings from the study and discuss them from the perspective of the resilience of the health system and also in comparison to other countries’ experiences in order to draw lessons and identify evidence gaps. However, it is important to first reflect on the limitations of the study. We acknowledge that despite the careful and ethical approach and the fact that questions remained focused on the professional or community experiences of participants (rather than their personal ones), the topic remains of high sensitivity, especially in the insecure and volatile context of Myanmar. This might have led to less open and transparent responses at times. However, researchers involved in data collection are well established and trusted in Myanmar and were able to create an environment of trust between interviewers and interviewees for the safety of all. In addition, we deliberately did not include any government representatives in the interviews. This limited the views and perspective gathered in the data collection process, but was required by a strict non-engagement policy with the current military government. Finally, the choice of one of the township-level study sites had to be modified due to security reasons. While ensuring the safety of the research team is imperative, we note that this somewhat reduced the comparative power of our analysis.

Despite these limitations, the study offers important insights into the role of NSAs in Myanmar in terms of service provision and in relation to their engagement with the state, and how these have evolved and changed over time as the country navigated the democratic transition process and the most recent shocks and crises.

### NSAs’ contribution to health system resilience

Findings confirm our initial hypothesis that NSAs play an increased role in service provision during time of acute crisis, due to the disengagement of the state. This is in line with findings from other settings including Central African Republic, DR Congo, Haiti, Palestine, Somalia [1], Afghanistan [1, 44] and Syria [45]. In all those cases, one of the key features is that the crisis is related to social and political instability and the state is weak and contested (compared, for example, to natural disasters).

In Myanmar, the historically weak government control and low service provision capacity further reinforced the role of NSAs [46]. During the democratic transition, there were attempts to standardise and regulate service provision by NSAs, but the situation rapidly reverted to the previous one as soon as a new political crisis hit the country. During the latest crises from 2020 to 2021, NSAs played a significant role in maintaining some levels of health service provision despite the challenges. In this sense, it can be argued that they contributed to the resilience of the health system. However, to better frame their contribution and draw lessons for other settings, it is important to note that NSA is a broad label and NSA categories and their respective relevance is varied in different settings. NSAs such as EHOs are a specific feature of Myanmar, not least because of their state-like support structures, length of their operation through multiple crises and phases, and the high level of support and recognition that they have received over time from external actors [47]. In other settings, other NSAs play a similar role but have different features. One example is the extensive network of faith-based health facilities in the DR Congo, which emerged after more than 15 years of civil conflict and state disengagement from health care provision [48]. Initially running parallel to the public system, the Catholic health network has been increasingly *de facto* integrated in the public one [49]. Beyond EHOs, our findings point to the role of local NGOs and CSOs in Myanmar. The importance of community actors for maintaining service delivery and their contribution to the resilience of the health system is also highlighted in other settings, such as Sierra Leone and Lebanon [50, 51], as well as in recent conceptual work stressing the relevance of the resilience of “community health systems”, alongside and in connection with the resilience of the formal health system [52]. Finally, in most settings, the private for profit sector remains one of the most relevant NSA, although their contribution to health system resilience is less clear. With reference to Yemen, Afghanistan and North West Syria, authors note the limited availability of data and information (which was also the case for Myanmar) and the difficulties in successful engagement with private sector which, in order to avoid potential challenges in terms of equity, accessibility and quality of the services provided, requires stewardship capacity in often challenging circumstances and weak government structures [1, 45, 53, 54].

### Resilience capacities and strategies of NSAs

In addition to highlighting the contribution of NSAs to the resilience of the health system, our analysis further illuminates the resilience strategies that were adopted by NSAs in Myanmar, as well as the underlying resilience capacities that were in place in order to deploy the resilience strategies. In particular, the comparison between periods of democratic transition with the current (crisis) time gives insights on how the resilience capacities built overtime by NSAs to provide services in adverse circumstances have informed the response to the latest crisis. Findings (summarized in Table 3 and below) confirm that the absorption and adaptation (resilience) strategies are built on the past experiences of NSAs in terms of approaches to service provisions that work for them and the communities they serve.

**Table 3:** Overview of resilience capacities and strategies

Resilience capacities	Absorption	Adaptation	Transformation
Social Networks and Collaboration	Initial interruption of collaboration to preserve staff and organisational safety	Informal coordination mechanisms established, building on pre-existing practices and existing trust and established personal relations (including with public providers)	
Availability, capacity and motivation of Human Resources	Re-strengthened role of community-based, mobile volunteers for security reasons	To address staff shortages, task shifting and involvement of family carers. Use of NGO staff for national programme delivery. CMD workers now in border area supported training in those areas.	
Availability of physical (medicines, technologies) and financial resources	NSA providers had retained and could use buffer stocks – a practice remained from previous crises.	NSAs reverted to the local purchasing of essential commodities. Informal and private supply channels also used which had been built in previous phases (also with donor support) Donors’ flexibility in funding approaches. Diaspora support. Alternative banking arrangements (third parties or outside of country to avoid government scrutiny)	
Dedicated leadership and distributed control			Top-down, tight control and leadership actively *prevented* transformative strategies to be implemented (or led to halt in NSA service provision) by controlling funding and activities, and intimidating staff
Strategic and flexible use of multiple or novel pathways and resources	NSAs continued service delivery with a reduced focus on TB, HIV and MCH services (historically core of their engagement). Private GPs became the first line of contact	Adapting to new context by reverting to previous practices and modes of delivery (see detailed description in Findings)	

*Source* authors [Resilience capacities are identified based on the R4R Resilience Framework (Fig. 1)]

For example, while the ‘convergence’, regulation and standardization processes during the democratic transition had partially changed the way they operated, NSAs were quick to revert to the previous approaches in terms of modes of delivery and how to use or access resources, including human, financial as well as physical (medicines and supplies). We noted that variations in healthcare delivery approaches also depended on the area’s previous history in terms of insecurity and NSAs engagement, which shaped the local resilience capacities of the health system. Where there was a historical parallel system built due to previous conflicts (for example, in Karen and Kachin), these were much more resilient compared to the newly affected conflict areas such as Chin, Magway and Saggaing regions. Empirical evidence from other countries shows different approaches to ensuring resilience of service delivery in contexts where previous learnings of resilience capacities could not be leveraged in the same way as in the border areas of Myanmar. For example, in North West Syria, a networked approach supported by external partners helped to keep service delivery functional in the context of multiple NGOs, fragmented interventions and weakened local governance structures [55]. In Mali, the resilience strategies deployed by health staff in community health centres in face of insurgency conflict were basic and uncoordinated, affected by the chronic dysfunctional state of the health system even before the main shock [56].

The flexibility and support of donors (which was also grounded in previous experiences of limited collaboration with the government and support to NSAs) played an additional key role in the shift to previous practices. This is a relatively exceptional example as in most instances engagement with NSAs remains complex for international donor organisations that often focus on state-delivered services [57]. In Myanmar, the successful engagement with NSAs to channel funding during the latest crisis phase was aided by the history of funder links with NSAs, due to the political history of Myanmar, as well as by the flexibility of organisations, such as the Global Fund to work with NSAs.

Collaborations, which were initially suspended due to the general environment of mistrust and the necessity to protect staff, restarted through informal mechanisms which had existed before the democratic transitions, or built on personal links more recently established. The essential contribution to the health system resilience in Myanmar of the strong social capital of informal channels and trusted networks (such as religious and community-based groups) has already been noted in other studies, with reference to the response to the Cyclone Nargis in 2005 [46] and is similar to other settings such as the DR Congo [48]. Not dissimilarly, personal and institutional ties between EHOs and government providers that had been forged during the democratic transition and convergence period were also found to remain strong, thus supporting the resilience of the local health systems also after the 2021 coup [34]. For example, many former government staff who joined the CDM moved to ethnic areas to work with EHOs colleagues and organisations. in addition to the resilience and resilience strategies of individual health workers [58], these connections helped preserve gains in service delivery and referrals despite the sequential crises [34]. This might have changed the geographic balance of health access across regions where previously it was better in the centre, while it is now possibly better in border areas.

While strategies of absorption and adaptation were noted in the study, we did not identify any transformation. This might point to the difficulty for NSAs to introduce radical changes when subjected to multiple (acute-on-chronic) shocks and a hostile political environment. For example, the ever-shifting requirements for registration with the MoHS, the control through the banking system as well as the security threats to staff have been mentioned as a major barrier to radical transformations in NSA service delivery approaches and roles.

## Conclusions

NSAs have historically played an important role in health service delivery in Myanmar. This study reviews how their role has evolved overtime, including reflecting on the growing engagement of the public sector with NSAs during the democratic transition period, and the changed approach and relations since the 2021 coup.

Similarly to what emerges from empirical evidence in other fragile settings, in Myanmar we note that health service provision increasingly relies on NSAs at a time of crisis, when the state is weaker and less trusted. Importantly, NSAs are shown to have built resilience capacities such as the flexibility and adaptability in modes of service delivery, supply chains, funding, communication and monitoring systems as well as health workers management, grounded in their experiences during previous crises. These resilience capacities and strategies, and their (relatively rapid) re-enactment at the time of the latest crisis, are shown to form the basis for supporting health service provision in adverse circumstances, and strengthening the overall resilience of the health system, despite the challenges created by the openly hostile environment.

While the study highlights resilience capacities and strategies that are specific to NSAs in Myanmar, some findings are in line with those in other settings affected by weak governance or contested governments, thus contributing to ongoing debates and offering potentially useful lessons on the role of NSAs in strengthening health system resilience in fragile states. Future research could explore this topic, with a focus on “state-less” contexts or those where governance is extremely weak, to highlight other common patterns, but also the diversity in resilience strategies which is to be understood and documented in order to identify suitable and tailored approaches to support and foster the contribution of NSAs to health system resilience in different settings.

Additionally, our findings contribute to global health governance debates, highlighting the need for international health policies and frameworks to more fully recognize and integrate the contributions of NSAs and leverage the strengths of diverse actors, including local and ethnic organizations, in the design and implementation of health interventions. International organizations and donors should consider the potential of NSAs to act as key partners in strengthening health systems in fragile and conflict-affected settings. This could lead to more effective and sustainable health outcomes, as NSAs are often deeply embedded in local contexts and capable of navigating complex political environments.

## Electronic supplementary material

Below is the link to the electronic supplementary material.

Supplementary Material 1

## Data Availability

The datasets generated and/or analysed during the current study are not publicly available due to safety concern for participants and researchers. Some of the data might be available from the corresponding author on reasonable request.

## Abbreviations

CDM: Civil Disobedience Movement
COVID-19: Coronavirus disease 2019
CSO: Civil Society Organisation
EAO: Ethnic Armed Organisation
EHO: Ethnic Health Organisation
FGD: Focus Group Discussion
GP: General Practitioner
HCCG: Health Care Convergence Group
KII: Key Informant Interview
LSTM: Liverpool School of Tropical Medicine
MHSCC: Myanmar Health Sector Coordination Committee
MOHS: Ministry of Health and Sport
NGO: Non-governmental Organisation
NLD: National League for Democracy
NSA: Non-state actor
R4R: ReBUILD for Resilience
UK: United Kingdom
