# Supplementary material for: Assessing the role of non-state actors in health service delivery and health system resilience in Myanmar
Source: Int J Equity Health. 2024 Oct 24;23:221. doi: 10.1186/s12939-024-02292-3 (PMC11515364; doi:10.1186/s12939-024-02292-3)
Supplement: Supplementary file 1 — Supplementary Material 1 [file 12939_2024_2292_MOESM1_ESM.docx]

**Annex 1**: Topic guides

Tool 1: Key informant interviews with international donors and funders, international NGOs, local NGOs, CSOs, EHOs, former healthcare managers

**Topic guide**

**Objectives:**

- Understand non-state actors’ roles and positioning in relation with R4R resilience framework during the **2018-2020 period**
- Explore enablers and barriers in carrying out health service delivery by non-state actors
- Identify learn lessons relevant for the current phase (post-2021) characterised by COVID-19 and political and security crisis and conflict.

**Introduction:** Introduce the project, the scope of the interview – see also Participant Information Sheet

**Informed Consent Process:** Ensure participant has read the information sheet, ask if they have any questions or areas for clarification, explain about confidentiality including recording the interview and voluntary consent to participate in the interview, complete consent sheet.

| **Information about the interviewee** | | | |
| --- | --- | --- | --- |
| Interviewee ID |  | Job title and cadre of interviewee |  |
| Age |  | Gender | Male □ Female □ Other □ |
| Place of work (organization name) |  | No of years working in this organization |  |
| Years of experience working in health sector |  | Level (international, national, district) |  |
| Name of interviewer |  | Date of Interview |  |
| Time of start of interview |  | Time of end of interview |  |

*Note: not all questions will be asked to each participant; but selected questions from this guide will be asked based on their role and experience.*

**Ice breaker**

- What is your current professional role? What is your professional relation/knowledge of non-state health actors*?

** Reminder to interviewer: by non-state actors, we mean international and national non-governmental organisations (NGOs), civil society organisations (CSOs) and Ethnic and Community-Based Health Organizations (ECBHOs) that are engaged in directly providing health services to communities or in supporting/funding those health services. Respondents would need to focus their answers and discussions on the non-state actors/organisations they are familiar with. If more than one, helpful if they can draw comparisons.*

**Role of non-state providers of healthcare in the 2018-2020 period**

- What is the role that non-state health service provision has played in Myanmar, in particular with reference to the 2018-2020 period?
  - What services are non-state providers focusing on?
  - Which communities do they serve?
  - What approaches to service delivery have they privileged, and why?
  - What gap do non-state actors fill in, within the broader health system?
  - What do you think are the community’s views on non-state health service provision? [Do they use non-state health providers? Do they value them? And why?]
- How does the role of non-state health providers differ, or how has it evolved, compared to the previous years (i.e., pre-2015)?
- What were the main challenges or barriers that non-state actors providing health services during the 2018-2020 period faced?
- Conversely, what have been the enablers to non-state health service delivery during the 2018-2020 period?
- How has non-state health service delivery adapted during the initial phases of the COVID-19 pandemic? [ we are interested here in the period up to the end of 2020].
  - Did their service delivery model change, and how? (for ex, disease control, MCH prevention to clinical care, or clinics to mobile clinics, community work, etc.)
  - Did their target service users change, and why?
  - Did they have to change place of service delivery and why?

**Relation between non-state actors and government**

The period between 2018 and 2020 was important in terms of how government and non-state actors have started engaging with one another with the aim of reducing the fragmentation of the health system.

- Please reflect on that process: what do you think were the main achievements? What were the barriers to progress in integration/reduction of fragmentation?
- What were the main mechanisms in place for the non-state actors to engage with the government and the community?
- What is now left of that process? What can be still build on? Are there any lessons learned?

**Support to non-state provision of healthcare from external actors**

- What approaches has the international community taken to support non-state healthcare provision, during the 2018-2020 period? How these approaches shifted in the 2018-2020 period compared to previous periods (e.g. pre-2015)?
- How have levels of funding and their allocation to non-state healthcare providers from international actors changed over this period? (e.g. which type of providers/services/geographical areas have they focused on?).

**Non-state provision of healthcare and health system resilience**

*By resilience, we mean the (health) system’s ability to absorb, adapt and/or transform in response to shocks and stresses. The absorption/adaptation/transformation is done through certain pathways and using specific resilience capacities, which are what we want to better understand here.*

- Have non-state health providers contributed to building the resilience of the health system in Myanmar, in your view? How and why (or why not)?
  - Probe any challenges and adaptations they made for health financing
  - Probe any challenges and adaptations they made for health staff, including CHWs
  - Probe any challenges and adaptations they made for supplies and medicines
  - Probe any challenges and adaptations they made for governance, including partnerships and collaboration
  - Probe any challenges and adaptations they made for planning, reporting and information systems
  - Probe any challenges and adaptations they made for working with communities, especially vulnerable groups

**Final reflections**

- What do you think has worked well and less well in terms of non-state health provision in Myanmar? For instance, do you have examples of resources or approaches being used in a particularly strategic and flexible way to ensure service delivery and health system strengthening (or conversely, examples where they were not used strategically)?
- What are the main strengths and the main weaknesses of non-state health provision?
- How has the system been able to manage shocks through non-state provision of health services?
- Any reflections on the implications of those lessons in the current context in Myanmar?

END OF INTERVIEW

THANK THE RESPONDENT

Tool 2: Key informant interviews with INGO staff, local NGO staff, CSO representatives, EHO representatives and administrative health professionals working for non-state actors at district level in the selected sites.

**Topic guide**

**Objectives:**

- Understand non-state actors’ shifting roles and practices during the **post-2021 period**, at district level in the selected sites.
- Explore enablers and barriers (including “residual resilience capacities from previous crises) in carrying out and adapting health service delivery by non-state actors
- Understand the effects of crisis and adaptation on equity, accessibility, and quality of service delivery

**Introduction:** Introduce the project, the scope of the interview – see also Participant Information Sheet

**Informed Consent Process:** Ensure participant has read the information sheet, ask if they have any questions or areas for clarification, explain about confidentiality including recording the interview and voluntary consent to participate in the interview, complete consent sheet.

| **Information about the interviewee** | | | |
| --- | --- | --- | --- |
| Interviewee ID |  | Job title and cadre of interviewee |  |
| Age |  | Gender | Male □ Female □ Other □ |
| Place of work (organization name) |  | No of years working in this organization |  |
| Years of experience working in health sector |  | Level (international, national, district – *if district, specify: ….*) |  |
| Name of interviewer |  | Date of Interview |  |
| Time of start of interview |  | Time of end of interview |  |

*Note: not all questions will be asked to each participant; but selected questions from this guide will be asked based on their role and experience.*

**Ice breaker**

- What is your current professional role, in relation to non-state health service provision or support to service provision*?
- Can you describe if/how your work has changed after February 2021?

** Reminder: by non-state actors, we mean international and national non-governmental organisations (NGOs), civil society organisations (CSOs) and Ethnic and Community-Based Health Organizations (ECBHOs) that are engaged in directly providing health services to communities or in supporting/funding those health services.*

**Changes in context and role/positioning of non-state providers after February 2021** [ building on elements of response provided during ice-breaking question]

- What are the main changes in context in relation to the political, economic and security situation that has affected your professional role and the way non-state providers work or are supported?
- How has the health sector situation changed? For example, looking at the influx/out flux of clinical staff due to displacement, destruction/targeting of facilities and infrastructure, changes and disruptions in supply chains, service delivery and HMIS.
- How has the role and support of the international community to *your non-organization providing/supporting health service delivery* changed after February 2021?
- How has *your organization’s* relationship with the government and with the formal/public health system changed after February 2021?
- How has *your organization’s* relationship with the community it is based in/it serves changed after February 2021? What drove those changes?

**Shifting practices of non-state providers of healthcare after February 2021** [ building on elements of response provided during ice-breaking question]

- How has health service delivery *by your non-organization* changed after February 2021? And why? Probes:
  - changes in terms service delivery modes and models, and place of delivery – for example, focusing on mobile clinics, targeting certain groups, task shifting and/or increasing use of community health workers, etc. [ Why where these changes introduced? ]
  - changes in disease/service focus – for example, more focus on COVID-19 or other specific disease/services, less focus on others, etc. [ Why where these changes introduced? ]
  - funding levels and funding flows – for example, reduction in funding, changes in sources of funding, delay in funding disbursement, etc. [ What drove these changes?]
  - could you mention other examples where you can see service delivery or other elements of your work were adapted or transformed to respond to challenges due to the crisis?
- What does this imply in terms of equity, accessibility, and quality of service delivery of different types of services (COVID-19, humanitarian services, routine health services, etc.)? Probe:
  - Are you reaching the same number of patients/service users? Did you see a decrease or increase in visits? For all services, or for some specifically? Why?
  - Are services accessible for all population groups or do you see some being not able to use it? Have you managed to target and provide services to vulnerable groups? What adaptations have you put in place to ensure accessibility and equity of service delivery?
  - Have you been able to maintain the same quality of services or not? What were the main challenges to do so? (i.e., lack of staff, issues with drug supply, difficulties in supervision, lack of funds, etc). How did you overcome these challenges?
- Are there any lessons learned on how to deal with crises by absorbing shocks, and adapting or transforming health service provision or other aspects of your work, that you have learned from previous periods and shocks, and you have put to practice during this crisis? Can you provide some examples of the lessons learned in the past and how they were applied more recently?
  - Probe any lessons learnt on getting and managing funds
  - Probe any lessons learnt on staffing, including community staff and volunteers
  - Probe any lessons learnt on management of supplies, medicines and infrastructure
  - Probe any lessons learnt on management, partnerships and collaboration
  - Probe any lessons learnt on planning and reporting
  - Probe any lessons learned on working with communities, especially vulnerable groups
- What is your experience of the support of international community to non-state actors? Do you have any specific recommendations to them?
- Do you have any final thoughts, reflections or recommendations?

END OF INTERVIEW

## Tool 3: in-depth interviews with health workers working for non-state providers, in the selected sites. Includes: health workers working for INGOs, local NGOs, CSOs, EHOs, local volunteers and CHWs

**Topic guide**

**Objectives:**

- Understand views and perspectives on the shifting roles and practices of non-state actors during the **post-2021 period**, at district level in the selected sites.
- Understand how the experience and practice of health workers working for non-state actors has changed overtime, and in particular with reference to the post-2021 period.
- Understand the effects of crisis and adaptation on equity, accessibility, and quality of service delivery
- Identify main challenges, coping mechanisms and lessons learned on adapting to shocks to carry out equitable and quality service delivery, from the perspective of (frontline) non-state health workers.

**Introduction:** Introduce the project, the scope of the interview – see also Participant Information Sheet

**Informed Consent Process:** Ensure participant has read the information sheet, ask if they have any questions or areas for clarification, explain about confidentiality including recording the interview and voluntary consent to participate in the interview, complete consent sheet.

| **Information about the interviewee** | | | |
| --- | --- | --- | --- |
| Interviewee ID |  | Job title and cadre of interviewee |  |
| Age |  | Gender | Male □ Female □ Other □ |
| Place of work (organization name) |  | No of years working in this organization |  |
| Years of experience working in health sector |  | Level (*specify district*) |  |
| Name of interviewer |  | Date of Interview |  |
| Time of start of interview |  | Time of end of interview |  |

*Note: not all questions will be asked to each participant; but selected questions from this guide will be asked based on their role and experience.*

**Ice breaker**

- Can you describe your current professional role and the non-state organization your work with? What is your/your organisation’s role in relation to non-state health service provision or support to service provision*?

** Reminder: by non-state actors, we mean international and national non-governmental organisations (NGOs), civil society organisations (CSOs) and Ethnic and Community-Based Health Organizations (ECBHOs) that are engaged in directly providing health services to communities or in supporting/funding those health services.*

**Brief professional “life history” of health worker**

- Looking back in time, can you tell me briefly about your professional career, from training/ qualification up to your current role? [Probe]
  - What is your qualification, when/where do you obtain it?
  - What professional roles have you held in the past? For which organisations?
  - What led you to work in this role?
- What are the main *personal* challenges that you have faced professionally overtime? For example, in relation to your personal security, your income, hours of work, career progression, etc.
- How did you navigate them or cope with them? [ e.g., support from family and community, support from employer and colleagues, etc.] Do you feel you have enough support or would like to be further supported, and how/by whom?
- What is your relationship with the community you work in/for?
- What motivates you to work every day?
- **Changes in context and adaptations to challenges Can you tell me about what your organization do for the community and its main focus in health service delivery.**
- Focusing on the recent political and health crises (after February 2021), can you describe if/how your work has changed after February 2021?
- What are the main changes in context in relation to the political, economic, COVID 19 and security situation that has affected your professional role and the way your organisation works?
- How has the health sector situation changed? For example, looking at the influx/out flux of clinical staff due to displacement, destruction/targeting of facilities and infrastructure, changes and disruptions in supply chains, service delivery and HMIS.
- How has *your organization’s* role changed after February 2021?
- How has *your organization’s* relationship with the community it is based in/it serves changed after February 2021? Why did it change (if it did)?

**Shifting practices of non-state providers of healthcare after February 2021**

- How has health service delivery *by your non-organization* changed and adjusted after February 2021? And why? Probes:
  - changes in terms service delivery modes and models, and place of delivery – for example, focusing on mobile clinics, targeting certain groups, task shifting and/or increasing use of community health workers, etc. [ Why where these changes introduced?]
  - changes in disease/service focus – for example, more focus on COVID-19 or other specific disease/services, less focus on others, etc. [ Why where these changes introduced?]
  - funding levels and funding flows – for example, reduction in funding, changes in sources of funding, delay in funding disbursement, etc.
  - could you mention other examples where you can see service delivery or other elements of your work were adapted or transformed to respond to challenges due to the crisis?
- What are your views on these changes? Did they address the challenges effectively? How did they work in practice for you?
- What does this imply in terms of equity, accessibility, and quality of service delivery of different types of services (COVID-19, humanitarian services, routine health services, etc.)? Probe:
  - Are you reaching the same number of patients/service users? Did you see a decrease or increase in visits? For all services, or for some specifically? Why?
  - Are services accessible for all population groups or do you see some being excluded? Have you managed to target and provide services to vulnerable groups? What adaptations have you put in place to ensure accessibility and equity of service delivery?
  - Have you been able to maintain the same quality of services? What were the main challenges to do so? (i.e., lack of staff, issues with drug supply, difficulties in supervision, lack of funds, etc). How did you overcome these challenges?

**Learning from the past to better navigate current crisis, reflections and recommendations**

- Looking back at the past (before February 2021) [to be adapted to the respondent’s specific experience], what are the main challenges that you or your organisation have faced *in relation to the context* in which you work? (e.g. previous political crises, insecurity, epidemics, lack of funds, shortages of drugs or staff, etc.).
  - How did you and your organisation manage to address them?
  - What has your organisation learned from addressing those challenges over time? For instance, these could be lessons learned on how to quickly change service provision modes (from clinic to mobile clinic) or service focus, or how to re-deploy staff, or how to maintain communication with staff in the frontlines.
  - For you personally, are there any lessons on coping mechanisms, on finding support at community level or from colleagues, creating communication channels, etc.
  - Have these lessons helped you personally and/or your organisation to navigate this most recent crisis?
- Do you have any final thoughts, reflections or recommendations, thinking about your and your organisation’s work in the future?

END OF INTERVIEW

THANK THE RESPONDENT

Tool 4: in-depth interviews with community members/service users working for non-state providers in the selected sites

**Topic guide**

**Objectives:**

- Gather the views and perspectives of users and communities on the roles and practices of non-state actors, at district level in the selected sites.
- Understand the experience of service users and community members in relation to services provided by non-state actors and how this has changed overtime, and in particular with reference to the **post-2021 period**.
- Understand the effects of crisis and adaptation on equity, accessibility, and quality of service delivery, from the perspective of service users and community members.

**Introduction:** Introduce the project, the scope of the interview – see also Participant Information Sheet

**Informed Consent Process:** Ensure participant has read the information sheet, ask if they have any questions or areas for clarification, explain about confidentiality including recording the interview and voluntary consent to participate in the interview, complete consent sheet.

| **Information about the interviewee** | | | |
| --- | --- | --- | --- |
| Interviewee ID |  | Location |  |
| Age |  | Gender | Male □ Female □ Other □ |
| Name of interviewer |  | Date of Interview |  |
| Time of start of interview |  | Time of end of interview |  |

*Note: not all questions will be asked to each participant; but selected questions from this guide will be asked based on their role and experience.*

General health care seeking

- What kinds of health issues have you and your family faced in the past 1-2 years?
- Where did you go for help?
- Why did you choose that provider?
- What was your experience with them?

**Non-state health service knowledge, use and views**

- Have you or your family used health services provided by NGO health providers? Which organization/provider and for what service needed?
- What are your experiences of NGO health providers?
  - Why do you, your family and other members of the community use those services?
  - Do you think they address the health needs of your community?
  - Are there specific groups that are not able to access to services provided by NGO health providers? Can you give me some examples?
- What are your general views on NGO health providers in terms of:
  - quality
  - price
  - waiting time
  - attention to patient’s needs
  - ability to serve vulnerable groups and the poorest
  - your trust in them as a provider

**Perceptions on changes in context and adaptations of service delivery practice**

- Focusing on the recent political and health crises, can you describe if/how it has affected your access to health services and your experience of health service provision?
  - What are the main difficulties in accessing the health services you need?
  - What are the main challenges in relation to services you are able to access? (for example, in relation to quality, price, waiting time, personal security, trust and confidence in the services, etc.)
  - What are the main strategies/approaches you have used to ensure you/your family can continue to access the health services you need?
- What is the role that NGO health providers have played in ensuring that you and your family can access services? Have you noticed any changes in how NGO health providers operate and deliver services?
  - Can you describe these changes? (e.g. relationship)
- What do you think of these changes?
  - Do they respond to the challenges due to the crisis?
  - Do they address your/your family’s health needs, and/or the health needs of the community?
  - Is quality of services affected? Why and how?
  - Is the accessibility of services affected? Why and how?
  - Do you think any particular groups are now excluded from health services and suffer particularly?

**Learning from the past to better navigate current crisis, reflections and recommendations**

- Looking back at the past, how do you think NGO health provider role has changed in your community?
- Do you have any final thoughts, reflections or recommendations?

END OF INTERVIEW

THANK THE RESPONDENT
